# Effects of Volatile Anaesthetics and Iron Dextran on Chronic Inflammation and Antioxidant Defense System in Rats

**DOI:** 10.3390/antiox11040708

**Published:** 2022-04-03

**Authors:** Dyana Odeh, Nada Oršolić, Emanuela Adrović, Lydia Gaćina, Petra Perić, Sahar Odeh, Vedran Balta, Nikola Lesar, Marina Kukolj

**Affiliations:** Division of Animal Physiology, Faculty of Science, University of Zagreb, Rooseveltov Trg 6, 10000 Zagreb, Croatia; eadrovic@stud.biol.pmf.hr (E.A.); lydia.gacina@uniri.hr (L.G.); pperic@stud.biol.pmf.hr (P.P.); spec.gin.ord.odeh@dzkkz.hr (S.O.); vedran.balta@skole.hr (V.B.); nikola.lesar@student.pmf.hr (N.L.); kukoljmarina@gmail.com (M.K.)

**Keywords:** sevoflurane, isoflurane, iron dextran, chronic inflammation, antioxidative/prooxidative effect, macrophages

## Abstract

Iron, as an essential microelement, is involved in cell proliferation, metabolism, and differentiation. It also modulates the fate and function of macrophages in hematopoiesis and macrophage-mediated inflammatory responses. On the other hand, anesthetics can affect the inflammatory process by modulating the response to stress or the functions of immune cells. The aim of this paper is to understand how excessive iron intake alters physiological, functional characteristics of peripheral tissues and whether different anesthetics can alter cell metabolism regarding oxidative stress (OS) and inflammation through regulation of macrophage polarization. Y59 rats were injected intraperitoneally with iron dextran solution at a dose of 50 mg/kg or were exposed to inhaled anesthetics sevoflurane and isoflurane and their combination for 28 days every other day. The results show that the use of anesthetics reduces the rat’s organ weight and increases OS in peripheral tissues, leading to M1 macrophage polarization. Excessive iron intake leads to increased OS, inflammation, and an increased ratio of IL-12/IL-10 cytokines to the M1 macrophage phenotype. Iron, in combination with sevoflurane, has a protective effect in tissues showing the M2 phenotype of macrophages. The combination of iron dextran and isoflurane in rats leads to an increase in the erythropoiesis process made possible through the induction of hypoxia.

## 1. Introduction

General anesthesia is one of the greatest achievements of medicine; it allows safe surgeries to be performed safely, even in very young or old patients. However, it is not without risk, and this is a fact that is accepted daily by a large number of patients [1]. Inhaled anesthetics are often used to introduce or maintain anesthesia, especially in more demanding and extensive surgeries. These are very volatile fluids that enter the body by breathing and then diffuse through the alveo-capillary membrane into the bloodstream [2]. They are then delivered through the bloodstream to the central nervous system (CNS) and other organs. Most of these anesthetics are removed from the body by respiration, but some are metabolized in the liver by the cytochrome oxidase P-450 family and excreted by the kidneys [1]. However, even today, not all mechanisms by which inhaled anesthetics perform anesthesia in animals and humans are fully elucidated despite their widespread clinical use. Previous research has shown that volatile anesthetics (VA) work by targeting multiple targets in the CNS. By studying the mechanisms of action of VA, they have been shown to act on caspase activation, increase β-amyloid protein production, increase proinflammatory cytokines, increase tumor necrosis factor (TNF-α), and support cell apoptosis [3].

Additional side effects and toxicity include nephrotoxicity, hepatotoxicity, cardiac arrhythmias, postoperative nausea and vomiting, respiratory depression and irritation, malignant hyperthermia, and post anesthesia agitation [4]. Inhaled anesthetics are not biologically inert compounds, and their metabolites can cause acute or chronic toxicity through several mechanisms: (1) intracellular accumulation of metabolites, (2) formation of haptens that can lead to hypersensitivity or immunoreaction, and (3) formation of reactive metabolites that can covalently bound cellular macromolecules and generate free radicals. Binding of reactive metabolites of inhaled anesthetics to tissue proteins may cause the formation of hapten–protein conjugates [1].

There is growing evidence to suggest the role of oxidative and nitrative stress in a potential adverse reaction to anesthetics that stimulate reactive oxygen species (ROS) production. Evidence suggests that oxidative stress is the primary cause of the pathogenesis of inflammation, partial ischemia, and metabolic diseases [5]. The main role of the inflammatory process is to protect the host from unwanted stimuli, such as pathogenic infections and mechanical stress. However, persistent inflammation and increased levels of oxidative stress lead to the accumulation of numerous inflammatory cells including macrophages, as main sources of cytokines, which participate in the regulation of various processes ranging from the regulation of local and systemic inflammation to cellular proliferation, metabolism, chemotaxis, and tissue repair [6].

Besides inflammatory effects, the anti-inflammatory effects of isoflurane and sevoflurane, the most widely used volatile anesthetics in clinical practices, have also been observed in humans. An increasing number of studies indicate the safety of isoflurane and sevoflurane, given oxidative stress and inflammatory response, in patients undergoing minor surgery [7]. However, meta-analyses need to be combined with data from patients undergoing more severe and multiple surgeries, including anesthetics, and aspects such as genetic predisposition and comorbidities should also be considered to determine the safety of isoflurane and sevoflurane with respect to oxidative stress and inflammatory response in these patients. This creates the need for new research on toxic and protective effects on organs and processes in organisms as well as the inflammatory response. However, for decades, controversial and contradictory results have emerged confirming the anti-inflammatory and pro-inflammatory results of anesthesia, thus requiring their closer investigation because the type and duration of general anesthesia are also thought to affect the extent of perioperative inflammation [8]. In addition to anesthetics, the presence of heavy metals can cause an additional problem. It should be noted that extensive surgery, the presence of tumors, and various forms of tumor therapy lead to anemia, which requires additional iron intake, which can lead to toxicity due to free iron in the blood [9,10].

On the other hand, environmental pollution and excessive metal intake endanger the health of humans and animals, leading to pathophysiological diseases associated with disruption of homeostasis of the whole organism. With the growth of industrialization, increasing environmental pollution, and the use of various products, human exposure to heavy metals has increased dramatically over the past 50 years. Although essential for the normal functioning of metabolism, metals such as iron, zinc, and copper can have negative effects if they are outside the normal range in the body [11,12]. Increased levels of iron in the body lead to an increase in oxidative stress and inflammation, which can seriously impair the function of peripheral tissues and organs [13]. Thus, increased amounts of heavy metals dysregulate cellular metabolism and lead to disturbances in essential metals, oxidative stress (OS), inflammation, macrophage polarization, immune suppression, and disturbances in the regulation and absorption of important micronutrients. Additionally, the accumulation of metals in tissues leads to damage of hematopoietic tissues, liver, kidneys, brain, etc., and consequently to diabetes, depression, neurodegenerative damage, infertility, and even cancer [14]. An example of such a metal is iron, which can affect more than a few hundred biologically important functions and cause many undesirable consequences in the body. OS caused by heavy metals is the result of a negative shift in the balance between ROS production and the ability of biological systems to easily counteract or repair ROS-induced damage [15]. Further, excess iron ranges from rare hereditary disorders to more common medical conditions associated with chronic blood transfusions. Based on the occurrence of pathological changes caused by metals and anesthetics, it is necessary to know the key mechanisms of disorders of cellular metabolism and immune response in order to prevent the resulting disorders of the organism and disrupt its integrity.

Given the lack of data on exposure to inhaled anesthetics in the presence of heavy metals, it is assumed that such exposure will lead to different prooxidative/antioxidative effects on certain tissues and organs, chronic inflammation and metabolic disorders, immune system disorders, endocrine and paracrine interaction leading to long-term tissue damage caused by inflammation, ROS, proinflammatory cytokines, cell death, cell necrosis or autophagy, and the occurrence of autoimmune and chronic disorders. Therefore, the aim of this study is to (a) investigate the effect of long-term use of inhaled anesthetics alone and/or combined with iron dextran on the antioxidant/prooxidative effect of peripheral tissues and organs by monitoring changes in oxido-reduction and functional status in liver, spleen, kidneys, and lungs; (b) investigate how different levels of ROS caused by constant intake of iron, and inhaled anesthetics, lead to immune system disorders connected with macrophages polarization as a heterogeneous cell population, their adaptation, and their response to various stimuli caused by disturbed metal homeostasis.

## 2. Materials and Methods

### 2.1. Chemicals

Sevoflurane (C_4_H_3_F_7_O; Mr = 200.005 g/mol) and isoflurane (C_3_H_2_ClF_5_O; Mr = 184.49 g/mol) from Baxter, Deerfield, IL, USA were used as inhaled anesthetics. Iron dextran (FeH_2_O_4_S; Mr = 153.92 g/mol; ~100 mg/mL) from Santa Cruz Biotechnology, Dallas, TX, USA was used in this study as heavy metal. Additionally, from other chemicals saline solution or 0.9% sodium chloride manufactured by B. Braun Adria d.o.o., Zagreb, Croatia was used in the experimental treatment of animals.

### 2.2. Preparation of Iron Dextran Solution

The iron dextran solution was prepared by dissolving FeH_2_O_4_S in a subchronic dose of 50 mg/kg in purified water (*aqua pro*), manufactured by B. Braun Adria d.o.o., Zagreb, Croatia.

### 2.3. Animals

Both sexes of inbred Y59 rats, 3 months old, obtained from the Department of Animal Physiology, Faculty of Science, were used in the study. The study was performed on a total of 60 Y59 rats divided into 6 groups of 10 animals each according to the treatment. Animals were fed with standard diet for laboratory animals (Standard Diet GLP, 4RF21, Mucedola, Settimo Milanese MI, Milano, Italy) with the availability of food and water *ad libitum*, and housing conditions were standard (daily rhythm of 12 h and 12 h of darkness, temperature 24 °C with controlled humidity).

The research was approved by the Ethical committee of Faculty of Science, University of Zagreb (approval code: 251-58-10617-19-57, date of approval: 23 January 2019) and was performed in accordance with ethical and legal principles valid in the Republic of Croatia (Law on Animal Welfare, NN 102/2017 [16]; Law on Amendments to the Law on Animal Welfare, NN 37/13 [17]; Regulation on the Protection of Animals Used for Scientific Purposes, NN 55/13 [18]); and according to the Guide for the Care and Use of Laboratory Animals, DHHS (NIH) Publ # 86-23, National Research Council [19].

### 2.4. Experimental Design and Doses

Animals were individually labeled and weighed (digital scales ABS 220-4, Kern Sohn, Germany) before the start of the experiment, as well as during the experiment, and were therefore classified into groups with approximately similar body weight (±10 g). Based on the mean body weight of the animals per cage for each group, the number of individual preparations given during the experiment was determined. Animals were weighed at the beginning of the experiment, every seven days during the experiment, and on the day of sacrifice.

Pathophysiological changes were induced by intraperitoneal (*ip*) injection of iron dextran solution in a subchronic dose of 50 mg/kg and/or by exposing rats to inhaled anesthetic—Sevoflurane (2.4 vol %) and Isoflurane (1.3 vol %) [20] in a continuous oxygen flow of 3 L/min for 28 days (Supplementary Material, Table S1). The stated volume percentages ensure complete anesthesia of the animals and non-response to stimuli. The experimental animals were divided into 6 groups of 10 animals: group 1 served as a control group and received 0.9% NaCl every other day during the treatment. The second group of animals was exposed for two hours to 2.4 vol % inhaled anesthetic Sevoflurane during the treatment. The third group was exposed for two hours to 1.3 vol % inhaled anesthetic Isoflurane, and the fourth group received *ip* iron dextran solution. The fifth group of animals received 2.4 vol % inhaled anesthetic Sevoflurane two hours after *ip* injection of iron dextran solution, while the sixth group of animals received 1.3 vol % Isoflurane inhaled anesthetic two hours after *ip* injection of iron dextran solution during the treatment.

By exposing the animals to 28 days of treatment, the final or total dose of iron dextran was 600 mg/kg. The dose was selected based on the literature [13]. Immediately after the last treatment, the animals were anesthetized and analgesic *ip* with a combination of ketamine (Narketan^®^, Bioveta, Ivanovice na Hané, Czech Republic) at a dose of 75 mg/kg and xylazine (Xylapan^®^, Bioveta, Ivanovice na Hané, Czech Republic) at a dose of 10 mg/kg, and sacrificed to collect the blood, tissues, and organs for further analysis.

### 2.5. Blood Sampling

After adequate anesthesia of the animals, blood samples were collected from the abdominal aorta (*A. abdominalis*) according to the guidelines of the Clinical Laboratory Standards Institute [21] and the World Health Organization [22]. Thus, collected blood was used for biochemical, hematological, and spectrophotometric analyzes.

### 2.6. Estimation of Biochemical and Haematological Blood Parameters

For biochemical analysis, blood was collected in vacutainer without anticoagulants. To assess biochemical parameters, serum was used for further analysis. A Comprehensive Diagnostic Profile reagent rotor (VetScan^®^ kit) from Abaxis, York, UK was used for analysis, and biochemical parameters were analyzed on a VetScan^®^ VS2 device (Abaxis, York, UK). From serum (100 μL), the following biochemical parameters were determined: albumin (ALB), alkaline phosphatase (ALP), alanine aminotransferase (ALT), amylase (AMY), total protein (TP), globulin (GLOB), total bilirubin (TBIL), blood urea nitrogen (BUN), phosphorus (PHOS), creatinine (CRE), glucose, sodium, potassium, and calcium.

In vacutainer with the EDTA, blood samples were collected for analysis of haematological parameters. From haematological parameters, the total number of erythrocytes (Erc), the number of leukocytes (Lkc), differential blood count (DKS), haematocrit (Hct), haemoglobin (Hb), average erythrocyte volume (MCV), average content of haemoglobin in erythrocytes (MCH), average haemoglobin concentration in erythrocytes (MCHC), and erythrocyte size distribution (RDW) were determined. Haematological parameters were determined by the recommended analytical methods on the Horiba ABX169 electronic counter (Micros, Lille, France).

### 2.7. Animal’s Weight Change and Relative Organ Weight

After sacrifice, the liver, kidneys, spleen, and lungs were isolated and weighed on a digital scale (Analytical balance ABS 220–4, Kern and Sohn GmbH, Balingen, Germany). The obtained values of organ mass and animal mass were used to calculate the animal’s weight change and relative organ weight. The animal’s weight change and the relative organ weights were calculated according to the following formulas:

$$\mathrm{Percentage}\;\mathrm{change}\;\mathrm{in}\;\mathrm{weight}=\frac{\left(\mathrm{Final}\;\mathrm{weight}-\mathrm{Initial}\;\mathrm{weight}\right)\times 100}{\mathrm{Final}\;\mathrm{weight}}$$

$$\mathrm{Relative}\;\mathrm{organ}\;\mathrm{weight}\;\left(\frac{g}{100g}\right)=\frac{\mathrm{Total}\;\mathrm{organ}\;\mathrm{weight}\;\times 100}{\mathrm{Final}\;\mathrm{body}\;\mathrm{weight}}\;$$

### 2.8. Determination of Inflammatory Cytokines Level and Evaluation of Macrophage Polarization and Its Functional Activity

In order to determine the level of 12 cytokines and chemokines after administration of inhaled anesthetics, and iron dextran, the Rat Inflmmatory Cytokines Multi-Analyte ELISArray Kit (Qiagen, Hilden, Germany) was used and performed according to the manufacturer’s instructions. The absorbance values were read at 450 nm on Ao Absorbance Microplate Reader (Azure Biosystems, Dublin, CA, USA) with a wavelength correction at 540 nm.

The Griess reagent system (Promega, Madison, WI, USA) kit was used to measure nitric oxide. Nitric (II) oxide concentration was measured on homogenized samples of liver, kidney, and spleen, and 0.1 M sodium nitrite dissolved in water in concentrations from 0 to 100 μM was used as a standard. From the standard curve of the dependence of the absorbance on the nitrite concentration, the slope line was determined, and the concentration of NO_2_^−^ in the samples, expressed as μM/μL, was calculated via the slope line.

Arginase activity assay kit MAK112 (Sigma-Aldrich, St. Louis, MA, USA) was used to measure the level of arginase 1 activity from a kidney and spleen and was performed according to the manufacturer’s instructions. Arginase is an enzyme that catalyzes the conversion of L-arginine to L-ornithine and urea, whereby the produced urea reacts specifically with the substrate to produce a color proportional to the activity of arginase, and the absorbance was measured at 430 nm (Ao Absorbance Microplate Reader, Azure Biosystems, Dublin, CA, USA). One unit of arginase represents the amount of enzyme sufficient to convert 1 μM L-arginine to ornithine and urea per minute at pH 9.5 and 37 °C.

### 2.9. Estimation of Oxido-Reduction Status in Tissues

Liver, kidney, spleen, and lung tissue samples were used to measure oxido-reduction status and were prepared as described in our previous paper [23].

Protein concentration in liver, kidney, spleen, and lungs tissue samples was measured by the method of Lowry et al. [24]. Protein concentration in tissue samples was used to express the values of the measured oxidative stress parameters (malondialdehyde, catalase, total glutathione, and superoxid dismutase).

To determine the concentration of malondialdehyde (MDA), the method of derivatization of MDA with 2-thiobarbiturate acid (TBA) was used to form a red fluorescent complex MDA-(TBA)_2_ whose concentration was measured spectrophotometrically (Libra S22, Biochrom, Cambridge, UK) at a wavelength of 532 nm. Total MDA concentration was calculated using the extinction coefficient for MDA (ε = 1.56 × 10^5^/M cm).

The activity of catalase was determined by the Aebium spectrophotometric method [25]. Catalase activity was determined by a decrease of the H_2_O_2_ amount, and catalase activity was measured spectrophotometrically (UV-160, Shimadzu, Kyoto, Japan) at a wavelength of 240 nm for one minute and was calculated using the extinction coefficient of H_2_O_2_ (ε = 39.4 m/M cm).

Total glutathione and SOD activity were determined according to paper [26]. Regarding total glutathione, in brief, 20 μL of sample, 40 μL of 0.035 M HCl, and 40 μL of 10 mM 5–5′-dithiobis-2-nitrobenzoic acid (DTNB, Ellman’s Reagent) was added to each microplate well. The absorbance was measured at 412 nm (Microplate reader Model 550, Bio-Rad, Hercules, CA, USA). The activity of superoxide dismutase was determined by inhibition of cytochrome c reduction in the xanthine/xanthine oxidase system (XOD). Briefly, 25 µL of sample was mixed with 1.45 mL reaction solution (0.05 mM cytochrome c; 1 mM xanthin, mixed in a 10:1 (*v/v*) ratio with DTNB). To determine SOD activity, the percentage of inhibition of XOD activity (optimized reaction ratio Δ A/min ~0.025) was first calculated, while SOD activity was calculated from % inhibition and expressed as U/mg proteins.

### 2.10. Statistical Analysis

The data were presented as mean ± standard error (mean ± SE). Statistical analysis was performed using the STATISTICA 14 program (StatSoft, Tulsa, OK, USA). Using the Kruskal–Wallis nonparametric test, the data were analyzed, and data were considered significant at the level of *p* < 0.05. Further analysis of the differences between the groups was made by multiple comparison of the mean values of all groups. Some data are represented in a scatter dot plot using GraphPad Prism 7 software (GraphPad Software, Inc., La Jolla, CA, USA).

## 3. Results

### 3.1. Inhaled Anaesthetics and Iron Dextran Induce Organ-Specific Responses and Changes in Animal’s Body Weight

**Figure 1:**
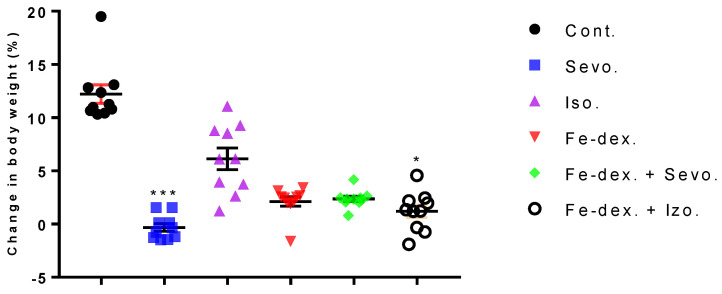
Dot plot of body weight change of rats treated with inhaled anesthetics, iron dextran, and their combination. Rats (*n* = 10 per group) were treated with *ip* injection of iron dextran (50 mg/kg) and exposed to 2.4 vol % sevoflurane inhalation and 1.3 vol % isoflurane inhalation, and to a combination thereof where inhaled anesthetics were given 2 h after *ip* injection of iron dextran. Animals were treated every other day for 28 days. Each animal sample is indicated by a single dot. The results are expressed as the mean value of each experimental group ± SE. * Statistically significant compared to the control group (* *p* < 0.05; *** *p* < 0.001). Abbreviations: Cont., control group; Sevo., sevoflurane group; Iso., isoflurane group; Fe-dex., iron dextran group; Fe-dex. + Sevo., group treated with iron dextran and sevoflurane; and Fe-dex. + Iso., group treated with iron dextran and isoflurane.

**Figure 2:**
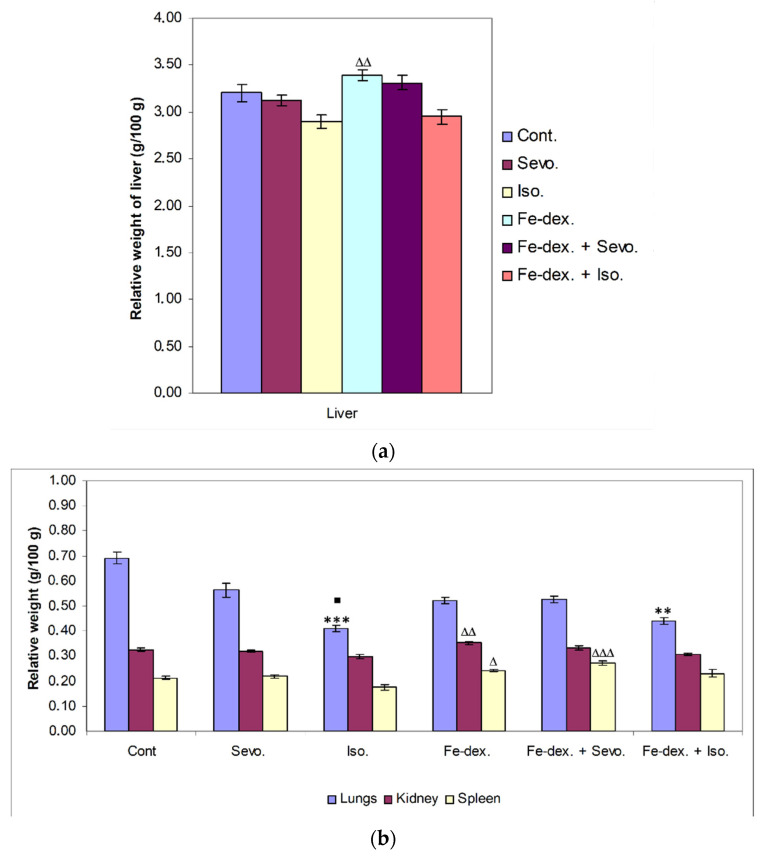
Relative weight of liver (**a**), lungs, kidney, and spleen (**b**) of rats treated with inhaled anesthetics, iron dextran, and their combination. Rats (*n* = 10 per group) were treated with *ip* injection of iron dextran (50 mg/kg), exposed to 2.4 vol % sevoflurane inhalation and 1.3 vol % isoflurane inhalation, and exposed to a combination thereof where inhaled anesthetics were given 2 h after *ip* injection of iron dextran. Animals were treated every other day for 28 days. The results are expressed as the mean value of each experimental group ± SE. * Statistically significant compared to the control group (** *p* < 0.01; *** *p* < 0.001); ^■^ Statistically significant compared to the sevoflurane group (^■^ *p* < 0.05); ^Δ^ Statistically significant compared to the isoflurane group (^∆^ *p* < 0.05; ^∆∆^ *p* < 0.01; and ^∆∆∆^ *p* < 0.001). Other statistically significant changes exist in: (**a**) liver—Fe-dex. vs. Fe-dex. + Iso. (*p* < 0.05); (**b**) kidney—Fe-dex. vs. Fe-dex. + Iso. (*p* < 0.05). Abbreviations: Cont., control group; Sevo., sevoflurane group; Iso., isoflurane group; Fe-dex., iron dextran group; Fe-dex. + Sevo., group treated with iron dextran and sevoflurane; and Fe-dex. + Iso., group treated with iron dextran and isoflurane.

In order to assess the possible inflammation among other parameters, we investigated the effect of inhaled anesthetics and iron dextran (Fe-dex.) on the body weight change and relative organ weight for 28 days of treatment (chronic effect). The results of the body weight change and relative organ weight of the liver, lungs, kidney, and spleen are shown in Figure 1 and Figure 2. The results of animal’s body weight indicate significantly lower body weight in the groups treated with sevoflurane (*p* < 0.001) and iron dextran + isoflurane (*p* < 0.05), compared to the control group (Figure 1).

Although there is no statistically significant change in the relative weight of the liver compared to the control group, there is a change in the other treatment groups. The relative weight of the liver in the group treated with iron dextran was statistically significantly higher compared to the groups treated with isoflurane (*p* < 0.01) and iron dextran + isoflurane (*p* < 0.05) (Figure 2a).

On the other hand, a statistically significant decrease in relative lung weight was observed in the groups treated with isoflurane (*p* < 0.001) and iron dextran + isoflurane (*p* < 0.01), compared to the control group (Figure 2b), while in the group treated with isoflurane it was also significantly decreased compared to the group treated with sevoflurane (*p* < 0.05). The relative weight of kidney was statistically significantly higher in the group treated with iron dextran compared to the groups treated with isoflurane and iron dextran + isoflurane. In the group exposed to isoflurane, a statistically significant decrease in the relative spleen weight was seen compared to the groups treated with iron dextran (*p* < 0.05) and iron dextran + sevoflurane (*p* < 0.001) (Figure 2b).

The increase in liver, spleen, and kidneys weight in the iron-dextran- and iron-dextran + sevoflurane-treated groups suggest that iron accumulation in these organs may induce tissue damage and inflammation with respect to iron toxicity, while a decrease in tissue weight in the isoflurane- and iron-dextran + isoflurane-treated groups leads to their accelerated decay.

### 3.2. Changes in Biochemical Parameters Are an Indicator of Tissue Damage after Rat Exposure to Inhaled Anesthetics and Iron Dextran

Confirmation of the inflammatory process was further assessed by measuring blood cell counts and biochemical parameters as indicators of tissue and organ damage. Tissue and organ damage were analysed by measuring the amount of circulating enzymes, proteins, metabolites, and substrates.

The results of enzyme and protein analysis are shown in Table 1, while results of metabolites and substrates are shown in Table 2.

During the treatment of animals with inhaled anesthetics and iron dextran, a statistically significant decrease in ALP concentration was seen in the groups treated with sevoflurane (123.67 ± 2.64; *p* < 0.05) and iron dextran + isoflurane (118.00 ± 7.96; *p* < 0.05), compared to the group treated with iron dextran (164.33 ± 9.12). The concentration of ALT was statistically significantly decreased in the groups treated with iron dextran, and iron dextran + sevoflurane, compared to the control group, and in the group exposed to isoflurane. The concentration of albumin and total proteins was statistically significantly lower in the groups treated with sevoflurane and iron dextran + isoflurane, compared to the group exposed to isoflurane, while the albumin concentration was also reduced and compared to the control group. Furthermore, the concentration of amylase was statistically significantly reduced in the group treated with iron dextran + sevoflurane, compared to the control group, and the groups exposed to sevoflurane and isoflurane, while the concentration of globulin was increased compared to the control group (Table 1).

Table 2 shows the largest changes in the concentration of metabolites and substrates in the groups treated with sevoflurane, isoflurane, and iron dextran + isoflurane during the 28 days of treatment. Concentration of blood urea nitrogen was significantly increased in the group exposed to sevoflurane compared to the iron dextran + sevoflurane-treated group (increased by 1.45×) and the control group (increased by 1.32×). Glucose was significantly increased in the groups treated with isoflurane and iron dextran + isoflurane, compared to the group exposed to sevoflurane, while in the group treated with iron dextran + isoflurane it was also increased compared to the control. In the group treated with iron dextran + isoflurane, the concentration of phosphorus (Fe-dex. + Iso. vs. Sevo.; *p* < 0.05) and potassium (Fe-dex. + Iso. vs. Fe-dex.; *p* < 0.001) was statistically significantly increased (Table 2). Sodium and potassium concentration were significantly increased in the group exposed to isoflurane compared to the group exposed to sevoflurane (*p* < 0.05), while potassium concentration was also significantly increased and compared to the iron dextran-treated group (*p* < 0.001).

Total bilirubin was not statistically significantly altered in any of the treated groups during the treatment and therefore is not shown in the Table 2.

### 3.3. Effect of Inhaled Anaesthetics and Iron Dextran on Haematological Parameters in Rats

**Figure 3:**
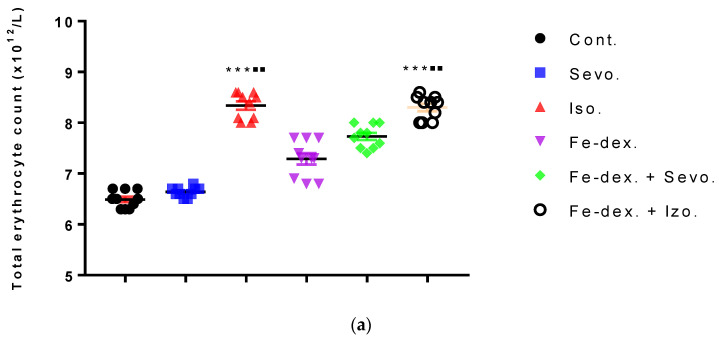

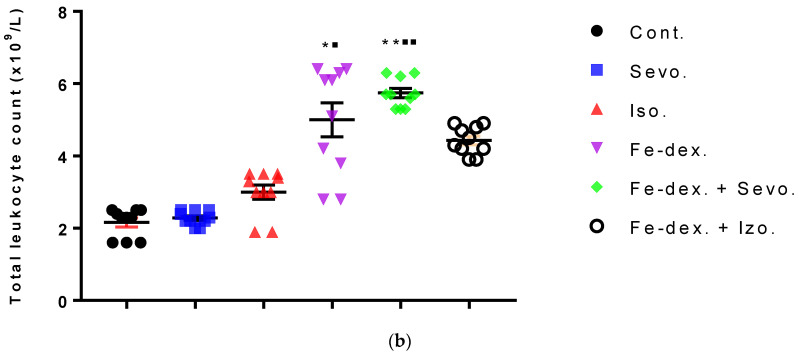
Total number of erythrocytes (**a**) and leukocytes (**b**) in the blood of rats treated with inhaled anesthetics and iron dextran and their combination. Rats (*n* = 10 per group) were treated with *ip* injection of iron dextran (50 mg/kg), exposed to 2.4 vol % sevoflurane inhalation and 1.3 vol % isoflurane inhalation, and exposed to a combination thereof where inhaled anesthetics were given 2 h after *ip* injection of iron dextran. Animals were treated every other day for 28 days. Each animal sample is indicated by a single dot. The results are expressed as the mean value of each experimental group ± SE. * Statistically significant compared to the control group (* *p* < 0.05; ** *p* < 0.01; *** *p* < 0.001); ^■^ Statistically significant compared to the sevoflurane group (^■^ *p* < 0.05; ^■■^ *p* < 0.01). Abbreviations: Cont., control group; Sevo., sevoflurane group; Iso., isoflurane group; Fe-dex., iron dextran group; Fe-dex. + Sevo., group treated with iron dextran and sevoflurane; and Fe-dex. + Iso., group treated with iron dextran and isoflurane.

**Figure 4:**
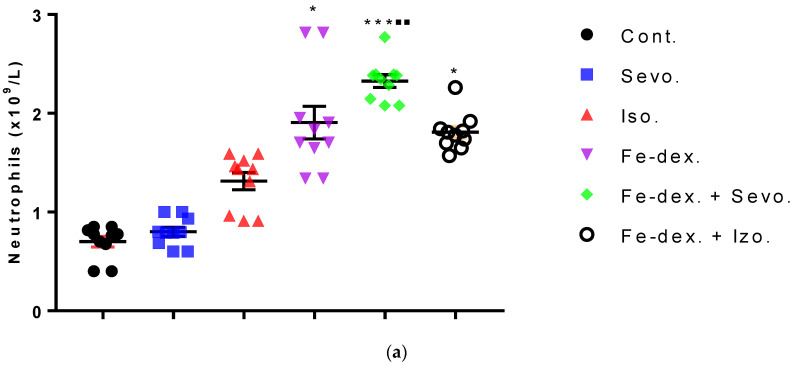

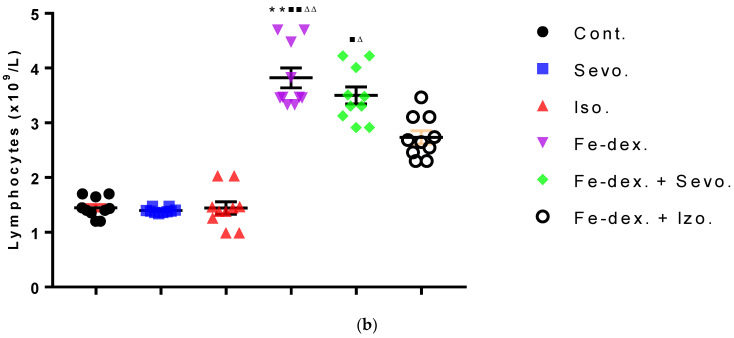
Total number of neutrophils (**a**) and lymphocytes (**b**) in the blood of rats treated with inhaled anesthetics and iron dextran and their combination. Rats (*n* = 10 per group) were treated with *ip* injection of iron dextran (50 mg/kg), exposed to 2.4 vol % sevoflurane inhalation and 1.3 vol % isoflurane inhalation, and exposed to a combination thereof where inhaled anesthetics were given 2 h after *ip* injection of iron dextran. Animals were treated every other day for 28 days. Each animal sample is indicated by a single dot. The results are expressed as the mean value of each experimental group ± SE. * Statistically significant compared to the control group (* *p* < 0.05; ** *p* < 0.01; *** *p* < 0.001); ^■^ Statistically significant compared to the sevoflurane group (^■^ *p* < 0.05; ^■■^ *p* < 0.01); ^∆^ Statistically significant compared to the isoflurane group (^∆^ *p* < 0.05; ^∆∆^ *p* < 0.01). Abbreviations: Cont., control group; Sevo., sevoflurane group; Iso., isoflurane group; Fe-dex., iron dextran group; Fe-dex. + Sevo., group treated with iron dextran and sevoflurane; and Fe-dex. + Iso., group treated with iron dextran and isoflurane.

Macrophages, as key parameters in the inflammatory process and iron homeostasis, recycle iron obtained by spent red blood cell (RBC) catabolism, thus maintaining a normal level of erythropoiesis and preventing anemia. Changes in haematological parameters are shown in Figure 3 and Figure 4, and Table 3.

Figure 3a shows an increase in the number of erythrocytes in the groups treated with isoflurane (8.23 ± 0.12) and iron dextran + isoflurane (8.32 ± 0.10), compared to the group exposed to sevoflurane (6.60 ± 0.04) and the control group (6.50 ± 0.07). In contrast, the number of leukocytes was increased in the groups treated with iron dextran (5.10 ± 0.73) and iron dextran + sevoflurane (5.77 ± 0.18), compared to the group exposed to sevoflurane (2.23 ± 0.09) and the control group (2.13 ± 0.17) (Figure 3b).

Differential blood count includes the number of neutrophils, lymphocytes, monocytes, and eosinophils. The total number of neutrophils was increased in the groups treated with iron dextran, iron dextran + sevoflurane, and iron dextran + isoflurane, compared to the control group, while in the group treated with iron dextran + sevoflurane it was increased and compared to the group exposed to sevoflurane (Figure 4a). Regarding the number of lymphocytes, its increase is visible in the groups treated with iron dextran and iron dextran + sevoflurane compared to the groups exposed to volatile anesthetics (Figure 4b). Additionally, in the group treated with iron dextran, this number was increased and compared to the control group. The number of monocytes and eosinophils did not change in any of the treated groups for 28 days of treatment and therefore is not shown in Figure 4.

The results of other haematological parameters are shown in Table 3. The greatest changes in other haematological parameters are visible in the groups treated with isoflurane and iron dextran + isoflurane. In the group exposed to isoflurane, the values of haemoglobin, haematocrit, and MCV were statistically significantly higher than in the control group, while the haemoglobin and haematocrit values were also higher than in the group exposed to sevoflurane. The concentration of MCHC was statistically significantly lower in the group exposed to isoflurane (8.38%) and in the group treated with iron dextran + isoflurane (10.81%), compared to the control group, while in the group treated with iron dextran + isoflurane was lower as well compared to groups treated with sevoflurane, iron dextran, and iron dextran + sevoflurane (Table 3). Besides, in the group treated with iron dextran + isoflurane, the haemoglobin value and the percentage of haematocrit and MCV were statistically significantly higher compared to the control group and the group exposed to sevoflurane, while the MCH value was significantly lower compared to the above-mentioned groups. Changes in the level of haemoglobin were also visible in the group treated with iron dextran + sevoflurane, where the level of haemoglobin was statistically significantly higher compared to the control group (18.68%; *p* < 0.05) and the group exposed to sevoflurane (16.88%; *p* < 0.05).

Summarizing the obtained results, it can be seen that long administration of isoflurane and iron dextran + isoflurane can induce hypotoxic microenvironment.

### 3.4. Inhaled Anaesthetics and Iron Dextran Lead to Different Macrophage Polarization and Influence the IL-12/IL-10 Axis Balance

Macrophages, as heterogeneous cells, are fully involved in resolving inflammation at different levels of the inflammatory process. They may be initiators of the inflammatory response and participate in its resolution and maintenance of homeostasis in the second step, through regulation of self-polarization after endogenous or exogenous stimulation or during different environmental conditions, and through reprogramming and continuous plasticity [27]. In normal tissue, the M1/M2 ratio of macrophages is highly regulated and increases during the inflammatory process, so in this paper the function of macrophages was examined by measuring nitric oxide parameters, arginase activity, and measuring inflammatory cytokine levels.

**Figure 5:**
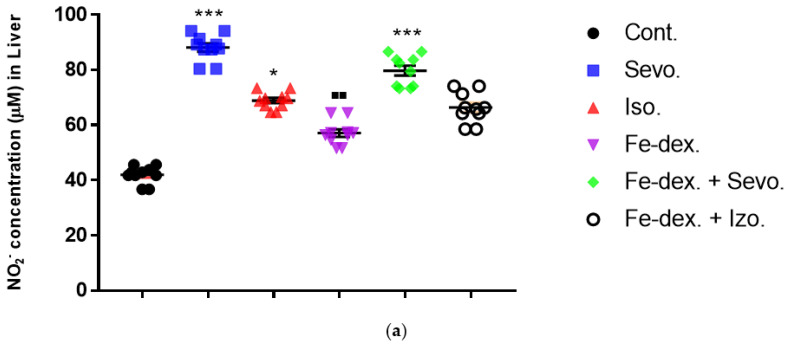

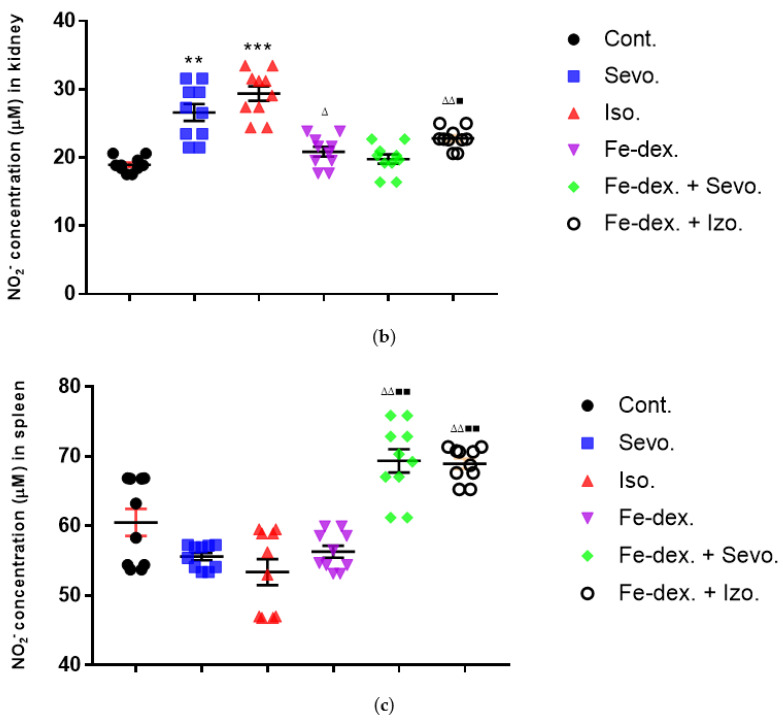
Effect of inhaled anesthetics and iron dextran on the nitric oxide (NO) activity in liver (**a**), kidney (**b**), and spleen (**c**) of rats treated for 28 days. Rats (*n* = 10 per group) were treated with *ip* injection of iron dextran (50 mg/kg), exposed to 2.4 vol % sevoflurane inhalation and 1.3 vol % isoflurane inhalation, and exposed to a combination thereof where inhaled anesthetics were given 2 h after *ip* injection of iron dextran. Animals were treated every other day for 28 days. Each animal sample is indicated by a single dot. The results are expressed as the mean value of each experimental group ± SE. * Statistically significant compared to the control group (* *p* < 0.05; ** *p* < 0.01; *** *p* < 0.001); ^■^ Statistically significant compared to the sevoflurane group (^■^ *p* < 0.01; ^■■^ *p* < 0.01); ^∆^ Statistically significant compared to the isoflurane group (^∆^ *p* < 0.05; ^∆∆^ *p* < 0.01). Other statistically significant changes exist in: (**a**) NO_2_^−^ concentration (µM) in liver—Fe-dex. vs. Fe-dex. + Sevo. (*p* < 0.05); (**c**) NO_2_^−^ concentration (µM) in spleen—Fe-dex. vs. Fe-dex. + Sevo. (*p* < 0.05); Fe-dex. vs. Fe-dex. + Iso. (*p* < 0.05). Abbreviations: Cont., control group; Sevo., sevoflurane group; Iso., isoflurane group; Fe-dex., iron dextran group; Fe-dex. + Sevo., group treated with iron dextran and sevoflurane; and Fe-dex. + Iso., group treated with iron dextran and isoflurane.

The results of nitric oxide (NO) activity in animals treated with inhalation anesthetics and iron dextran, and their combinations, are shown in Figure 5. The results show that the concentration of nitric oxide in liver of animals treated for 28 days was increased in the groups treated with sevoflurane (by 2.10×), isoflurane (by 1.65×), and iron dextran + sevoflurane (by 1.90×), compared to the control group (Figure 5a). Additionally, the concentration of NO in the liver was increased in the groups treated with sevoflurane (*p* < 0.001) and iron dextran + sevoflurane (*p* < 0.05) and compared to the group treated with iron dextran.

From the results of Figure 5b, it can be seen that the concentration of NO in the kidney tissue is increased in the groups treated with sevoflurane and isoflurane, compared to the control group and the group treated with iron dextran + sevoflurane. In addition, the group exposed to isoflurane showed a statistically significant increase in NO concentration (by 1.41×) compared to the group treated with iron dextran (Figure 5b).

Additionally, in the spleen tissue of animals treated for 28 days, a statistically significant increase in NO concentration was seen in the iron dextran + sevoflurane and iron dextran + isoflurane treated groups in relation to the groups treated with sevoflurane, isoflurane, and iron dextran (Figure 5c).

Macrophage biology is basically driven by the phenotype of macrophage arginine metabolism that is prevalent in ongoing immune response. Additionally, changes in nitric oxide and cytokines levels (Th2) are associated with L-arginine metabolism, which is essential for healing and maintaining healthy states, for example, by activating the immune system (macrophages), and these changes may disturb the L-arginine metabolism, usually by up- or downregulating arginase expression and activity, which may lead to chronic inflammations or other diseases. Thus, arginase generally acts as a dual factor that may cause different outcomes depending on the surrounding biochemical context and can cause pathological processes or a response mechanism to achieve homeostasis; therefore, we measured arginase activity in the kidney and spleen to assess their function.

**Figure 6:**
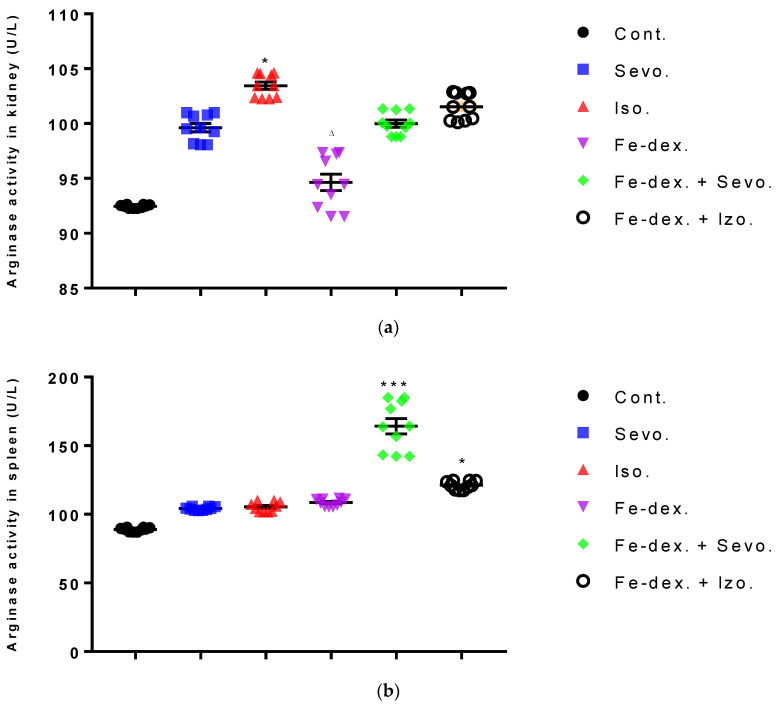
Effect of inhaled anesthetics and iron dextran on the arginase activity in kidney (**a**) and spleen (**b**) of rats treated for 28 days. Rats (*n* = 10 per group) were treated with *ip* injection of iron dextran (50 mg/kg), exposed to 2.4 vol % sevoflurane inhalation and 1.3 vol % isoflurane inhalation, and exposed to a combination thereof where inhaled anesthetics were given 2 h after *ip* injection of iron dextran. Animals were treated every other day for 28 days. Each animal sample is indicated by a single dot. The results are expressed as the mean value of each experimental group ± SE. * Statistically significant compared to the control group (* *p* < 0.05; *** *p* < 0.001); ^∆^ Statistically significant compared to the isoflurane group (^∆^ *p* < 0.05). Abbreviations: Cont., control group; Sevo., sevoflurane group; Iso., isoflurane group; Fe-dex., iron dextran group; Fe-dex. + Sevo., group treated with iron dextran and sevoflurane; and Fe-dex. + Iso., group treated with iron dextran and isoflurane.

The results of arginase activity in kidney tissue indicate its increase by 11.89% in the group exposed to isoflurane compared to the control group (*p* < 0.05) and the group treated with iron dextran (*p* < 0.05) (Figure 6a). In the sevoflurane-treated group and iron dextran + isoflurane-treated group, arginase activity in spleen tissue was increased compared to the control group (Figure 6b).

**Figure 7:**
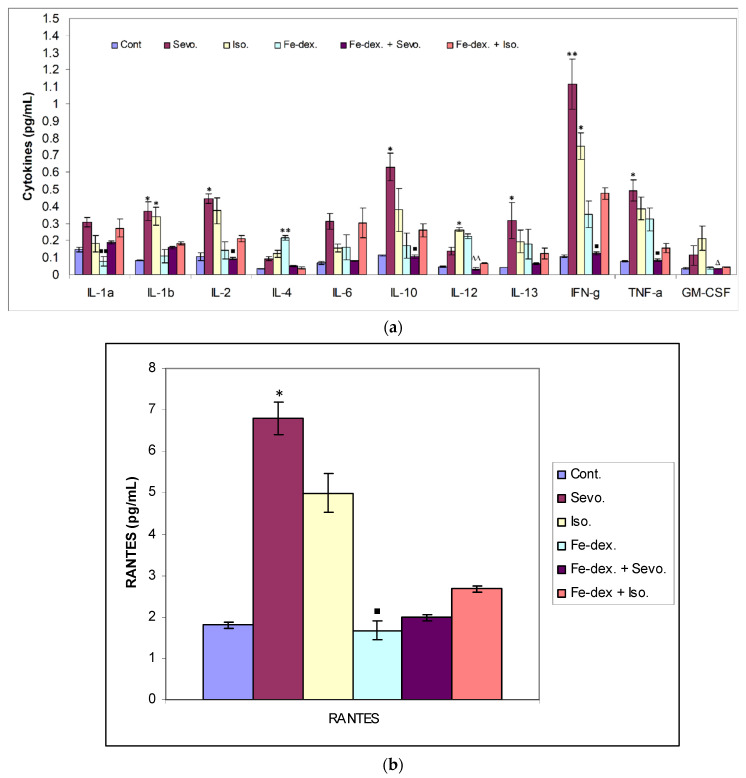
Effect of inhaled anesthetics and iron dextran on Th1, Th2 cytokines (**a**), and chemokine RANTES (**b**) in rats treated for 28 days. Rats (*n* = 10 per group) were treated with *ip* injection of iron dextran (50 mg/kg), exposed to 2.4 vol % sevoflurane inhalation and 1.3 vol % isoflurane inhalation, and exposed to a combination thereof where inhaled anesthetics were given 2 h after *ip* injection of iron dextran. Animals were treated every other day for 28 days. The results are expressed as the mean value of each experimental group ± SE. * Statistically significant compared to the control group (* *p* < 0.05; ** *p* < 0.01); ^■^ Statistically significant compared to the sevoflurane group (^■^ *p* < 0.05; ^■■^ *p* < 0.01); ^∆^ Statistically significant compared to the isoflurane group (^∆^ *p* < 0.05; ^∆∆^ *p* < 0.01). Other statistically significant changes exist in: (**a**) IL-4 (pg/mL)—Fe-dex. vs. Fe-dex. + Iso. (*p* < 0.05); IL-12 (pg/mL)—Fe-dex. vs. Fe-dex. + Sevo. (*p* < 0.05). Abbreviations: Cont., control group; Sevo., sevoflurane group; Iso., isoflurane group; Fe-dex., iron dextran group; Fe-dex. + Sevo., group treated with iron dextran and sevoflurane; and Fe-dex. + Iso., group treated with iron dextran and isoflurane.

Cytokines are secreted by cells of the immune system, and their primary role is mediation and regulation among other participants in the immune system. In addition, they participate in the body’s defences by causing protective local inflammation that can have a systematic impact at the acute level. A total of 12 inflammatory cytokines from rat serum treated with inhaled anesthetics, iron dextran, and their combinations were analysed, and the results are shown in Figure 7.

The greatest changes in cytokine levels were found in the group treated with sevoflurane, isoflurane, and iron dextran (Figure 7a,b). In the group exposed to sevoflurane, the levels of IL-1β, IL-13, and the chemokine RANTES were significantly increased than in the control group, while the levels of IL-2, INF-γ and TNF-α were significantly increased than in the group treated with iron dextran + sevoflurane and control group (Figure 7a,b), indicating a possible polarization of M1 macrophages. Figure 7a shows that in the group exposed to isoflurane, the level of IL-1β and INF-γ was increased compared to the control group, while the levels of GM-CSF and IL-12 were significantly increased compared to the group treated with iron dextran + sevoflurane. Moreover, IL-12 was also increased compared to the control group. In the iron dextran-treated group, IL-4 and IL-12 levels were significantly increased, while the IL-1α level was significantly lower than in the group exposed to sevoflurane (Figure 7a).

### 3.5. Oxidative Stress Parameters in Liver, Kidney, Spleen, and Lung Tissues in Rats Treated with Inhaled Anaesthetics and Iron Dextran

Increased amounts of iron dextran and large amounts of inflammatory mediators caused by chronic inflammation can lead to oxidative stress and reactive oxygen species (ROS), so this paper also monitors the parameters of oxidative stress in the liver, kidneys, lungs, and spleen.

**Figure 8:**
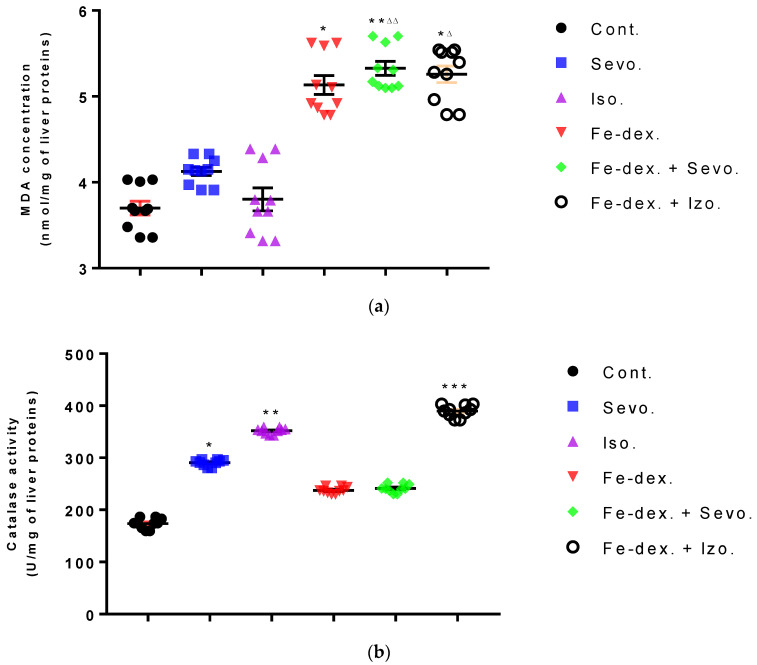

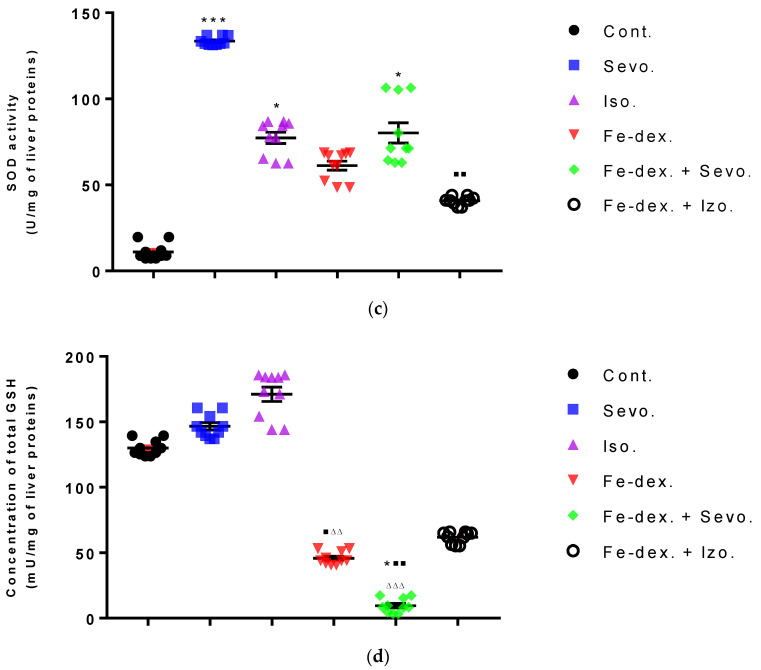
Effect of inhaled anesthetics and iron dextran on MDA (**a**), CAT activity (**b**), SOD activity (**c**), and GSH (**d**) in the liver of rats treated for 28 days. Rats (*n* = 10 per group) were treated with *ip* injection of iron dextran (50 mg/kg), exposed to 2.4 vol % sevoflurane inhalation and 1.3 vol % isoflurane inhalation, and exposed to a combination thereof where inhaled anesthetics were given 2 h after *ip* injection of iron dextran. Animals were treated every other day for 28 days. Each animal sample is indicated by a single dot. The results are expressed as the mean value of each experimental group ± SE. * Statistically significant compared to the control group (* *p* < 0.05; ** *p* < 0.01; *** *p* < 0.001); ^■^ Statistically significant compared to the sevoflurane group (^■^ *p* < 0.05; ^■■^ *p* < 0.01); ^∆^ Statistically significant compared to the isoflurane group (^∆^ *p* < 0.05; ^∆∆^ *p* < 0.01; and ^∆∆∆^ *p* < 0.001). Other statistically significant changes exist in: (**b**) catalase activity (U/mg of liver proteins)—Fe-dex. vs. Fe-dex. + Iso. (*p* < 0.01); Fe-dex. + Sevo. vs. Fe-dex. + Iso. (*p* < 0.05). Abbreviations: Cont., control group; Sevo., sevoflurane group; Iso., isoflurane group; Fe-dex., iron dextran group; Fe-dex. + Sevo., group treated with iron dextran and sevoflurane; and Fe-dex. + Iso., group treated with iron dextran and isoflurane.

Liver oxidative stress parameters showed an increase in MDA levels in the groups treated with iron dextran (by 38.48%; *p* < 0.05) and iron dextran + sevoflurane (by 43.90%; *p* < 0.01), compared to the control group (Figure 8a), while GSH levels were statistically decreased in these groups compared to the groups exposed to sevoflurane and isoflurane (Figure 8d). Catalase levels were increased in the sevoflurane and isoflurane groups compared to the control group (Figure 8b), while SOD levels were increased in these groups and in the iron dextran + sevoflurane-treated group, compared to the control group (Figure 8c). In the group treated with iron dextran + isoflurane, an increase in MDA and catalase levels is visible.

**Figure 9:**
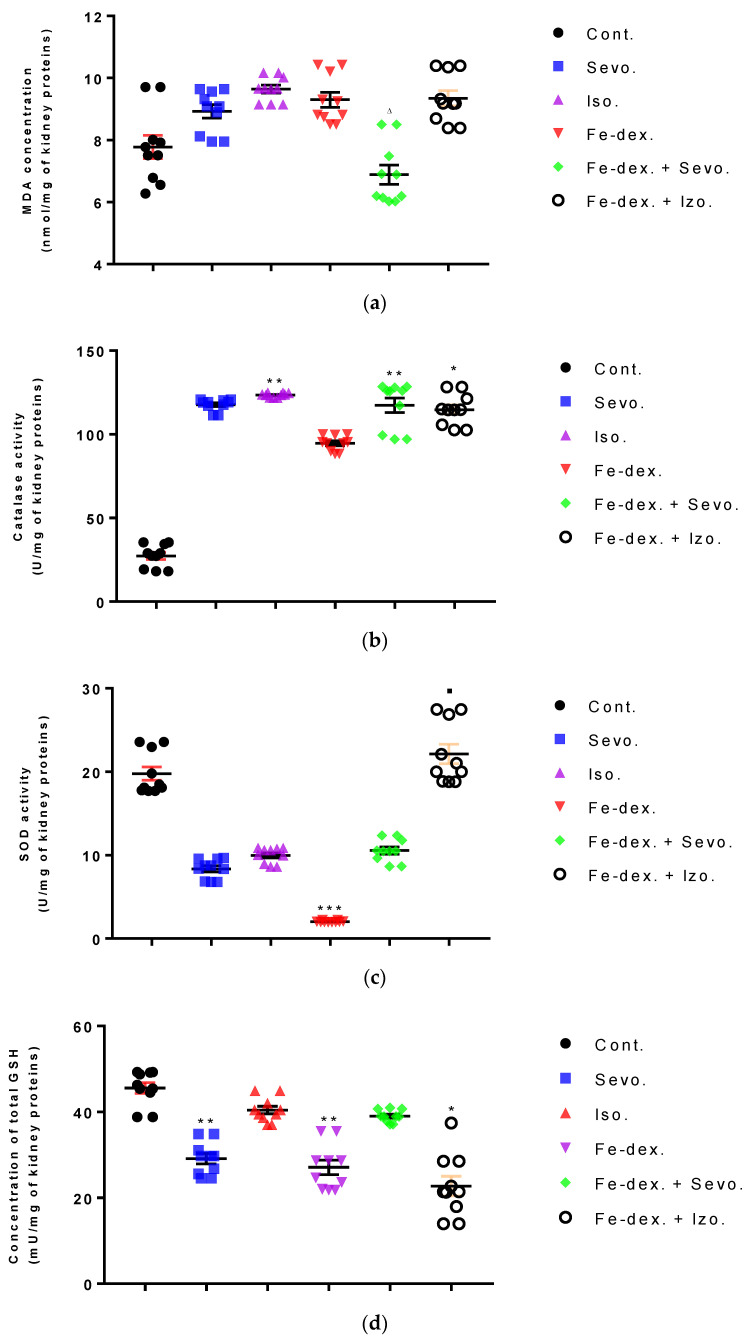
Effect of inhaled anesthetics and iron dextran on MDA (**a**), CAT activity (**b**), SOD activity (**c**), and GSH (**d**) in the kidney of rats treated for 28 days. Rats (*n* = 10 per group) were treated with *ip* injection of iron dextran (50 mg/kg), exposed to 2.4 vol % sevoflurane inhalation and 1.3 vol % isoflurane inhalation, and exposed to a combination thereof where inhaled anesthetics were given 2 h after *ip* injection of iron dextran. Animals were treated every other day for 28 days. Each animal sample is indicated by a single dot. The results are expressed as the mean value of each experimental group ± SE. * Statistically significant compared to the control group (* *p* < 0.05; ** *p* < 0.01; *** *p* < 0.001); ^■^ Statistically significant compared to the sevoflurane group (^■^ *p* < 0.05); ^∆^ Statistically significant compared to the isoflurane group (^∆^ *p* < 0.05). Other statistically significant changes exist in: (**c**) SOD activity (U/mg of kidney proteins)—Fe-dex. vs. Fe-dex. + Iso. (*p* < 0.001). Abbreviations: Cont., control group; Sevo., sevoflurane group; Iso., isoflurane group; Fe-dex., iron dextran group; Fe-dex. + Sevo., group treated with iron dextran and sevoflurane; and Fe-dex. + Iso. Group treated with iron dextran and isoflurane.

In the kidney tissue, an increase in MDA and CAT was seen in the group exposed to isoflurane (Figure 9a,b). Furthermore, the level of CAT was increased in the group treated with iron dextran + sevoflurane (by 4.26×; *p* < 0.01) and iron dextran + isoflurane (by 4.19×; *p* < 0.05), compared to the the control group (Figure 9b), while in the iron dextran + isoflurane treated group the GSH level was decreased compared to the control group (*p* < 0.05) (Figure 9d). In the groups treated with sevoflurane and iron dextran, the level of SOD was decreased compared to the group treated with iron dextran + isoflurane (*p* < 0.05; *p* < 0.001 respectively) (Figure 9c), while GSH level was decreased in these groups compared to the control group (*p* < 0.01) (Figure 9d).

**Figure 10:**
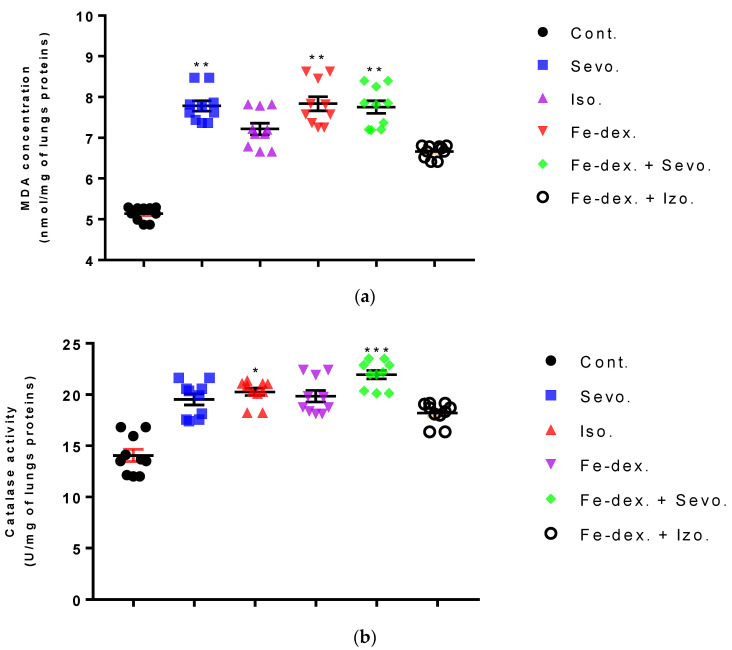

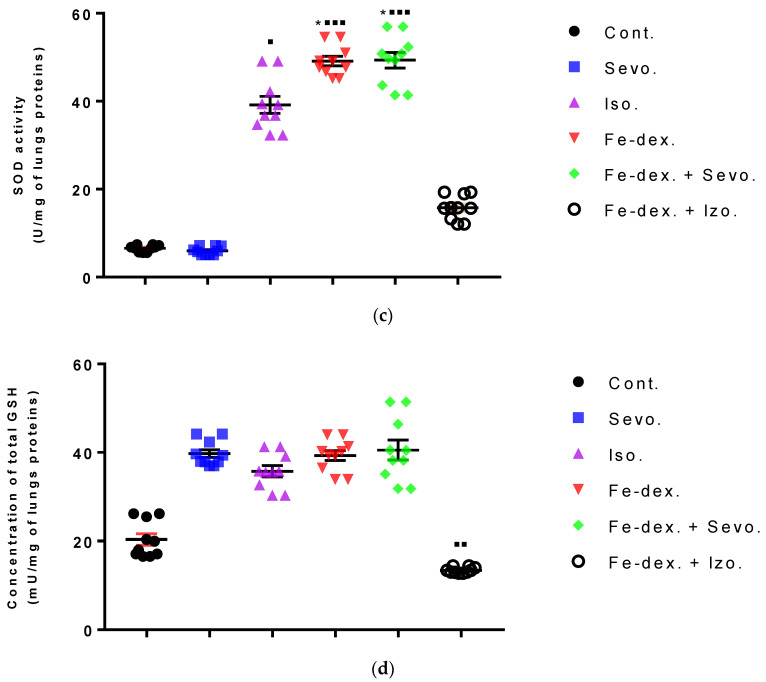
Effect of inhaled anesthetics and iron dextran on MDA (**a**), CAT activity (**b**), SOD activity (**c**), and GSH (**d**) in the lungs of rats treated for 28 days. Rats (*n* = 10 per group) were treated with *ip* injection of iron dextran (50 mg/kg), exposed to 2.4 vol % sevoflurane inhalation and 1.3 vol % isoflurane inhalation, and exposed to a combination thereof where inhaled anesthetics were given 2 h after *ip* injection of iron dextran. Animals were treated every other day for 28 days. Each animal sample is indicated by a single dot. The results are expressed as the mean value of each experimental group ± SE. * Statistically significant compared to the control group (* *p* < 0.05; ** *p* < 0.01; *** *p* < 0.001); ^■^ Statistically significant compared to the sevoflurane group (^■^ *p* < 0.05; ^■■^ *p* < 0.01; ^■■■^ *p* < 0.001). Other statistically significant changes exist in: (**d**) GSH activity (mU/mg of lungs proteins)—Fe-dex. vs. Fe-dex. + Iso. (*p* < 0.01); Fe-dex. + Sevo. vs. Fe-dex. + Iso. (*p* < 0.01) Abbreviations: Cont., control group; Sevo., sevoflurane group; Iso., isoflurane group; Fe-dex., iron dextran group; Fe-dex. + Sevo., group treated with iron dextran and sevoflurane; and Fe-dex. + Iso., group treated with iron dextran and isoflurane.

In the lungs of animals, an increase in MDA (by 1.52×; *p* < 0.01), CAT (by 1.64×; *p* < 0.001), and SOD (by 7.13×; *p* < 0.05) levels was seen in the iron dextran + sevoflurane group compared to the control group, while the SOD level was also increased by 11.62× compared to the group exposed to sevoflurane (Figure 10a–c). In the iron dextran-treated group, it can be seen from Figure 10a,c that the levels of MDA and SOD were increased compared to the control group (*p* < 0.01; *p* < 0.05, respectively). In animals exposed only to volatile anesthetics, an increase in MDA level (Sevo. vs. Cont.; *p* < 0.01) was seen in the lungs of animals exposed to sevoflurane, while an increase in CAT (Iso. vs. Cont.; *p* < 0.05) and SOD (Iso. vs. Sevo.; *p* < 0.05) levels was seen in animals exposed to isoflurane. In the lungs of animals treated with iron dextran + isoflurane, there was a decrease in GSH levels compared to the groups treated with sevoflurane, iron dextran, and iron dextran + sevoflurane (Figure 10d).

**Figure 11:**
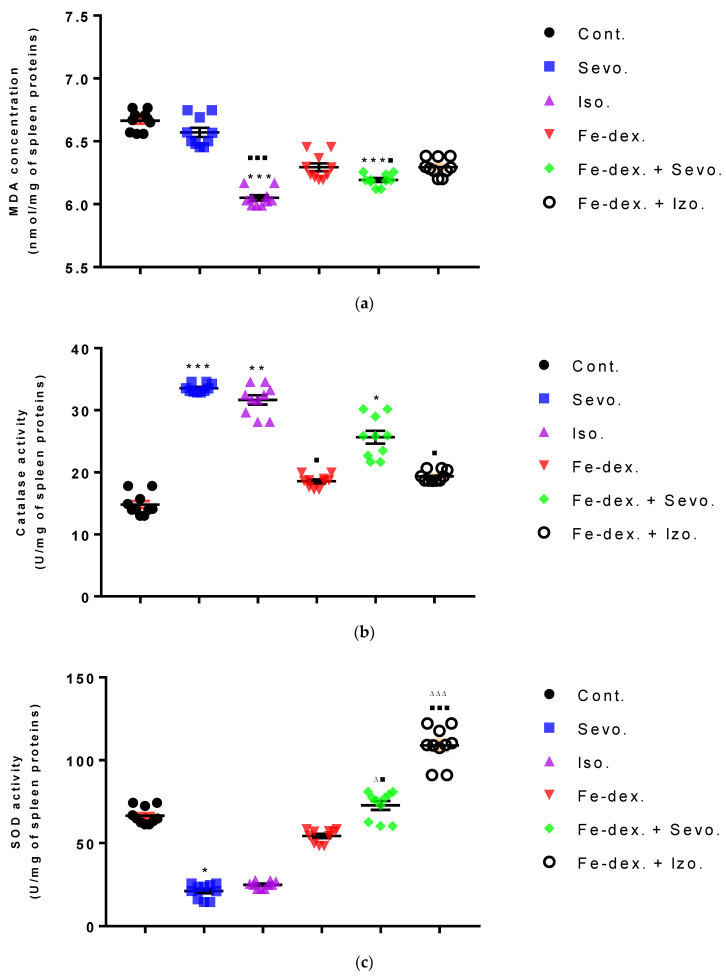

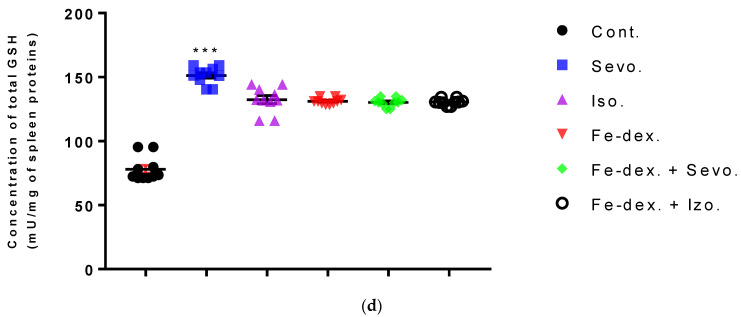
Effect of inhaled anesthetics and iron dextran on MDA (**a**), CAT activity (**b**), SOD activity (**c**), and GSH (**d**) in the spleen of rats treated for 28 days. Rats (*n* = 10 per group) were treated with *ip* injection of iron dextran (50 mg/kg), exposed to 2.4 vol % sevoflurane inhalation and 1.3 vol % isoflurane inhalation, and exposed to a combination thereof where inhaled anesthetics were given 2 h after *ip* injection of iron dextran. Animals were treated every other day for 28 days. Each animal sample is indicated by a single dot. The results are expressed as the mean value of each experimental group ± SE. * Statistically significant compared to the control group (* *p* < 0.05; ** *p* < 0.01; *** *p* < 0.001); ^■^ Statistically significant compared to the sevoflurane group (^■^ *p* < 0.05; ^■■■^ *p* < 0.001); ^∆^ Statistically significant compared to the isoflurane group (^∆^ *p* < 0.05; ^∆∆∆^ *p* < 0.001). Other statistically significant changes exist in: (**c**) SOD activity (U/mg of spleen proteins)—Fe-dex. vs. Fe-dex. + Iso. (*p* < 0.05). Abbreviations: Cont., control group; Sevo., sevoflurane group; Iso., isoflurane group; Fe-dex., iron dextran group; Fe-dex. + Sevo., group treated with iron dextran and sevoflurane; and Fe-dex. + Iso., group treated with iron dextran and isoflurane.

The spleen results of animals treated with inhaled anesthetics and iron dextran indicated a decrease in MDA levels in the group exposed to isoflurane compared to the group exposed to sevoflurane (*p* < 0.01) and control group (*p* < 0.001) (Figure 11a), while results of catalase levels indicated an increase compared to the control group (*p* < 0.01) (Figure 11b). In the group exposed to sevoflurane, the level of SOD was decreased by 3.15× (Figure 11c) and GSH was increased by 1.90× (Figure 11d), compared to the control group, while the level of CAT was increased compared to the groups treated with iron dextran, iron dextran + isoflurane, and the control group.

## 4. Discussion

None of the currently available halogen anesthetics are ideally adapted to their function, and their negative effects and dose-dependent toxicity have often been reported [2]. Many studies examine their potential hepatotoxic and nephrotoxic effects [28] because their end toxic metabolites are excreted by the kidneys, liver, or lungs using enzymes from the cytochrome oxidase family (CYP) [29]. The increased concentration of fluoride, which is considered a biomarker of nephrotoxicity and is as a final product of the metabolism of sevoflurane and isoflurane, has been shown to inhibit the natural antioxidant system and reduce defense against ROS. Fluoride has also been shown to stimulate ROS production in leukocytes, which is why some authors believe that sevoflurane is capable of producing OH•, O_2_^−^, and H_2_O_2_ and contributing to lipid peroxidation in many tissues and organs [8,30]. However, for decades, controversial results have emerged confirming the pro-inflammatory effects of anesthesia, as well as the anti-inflammatory effects on animal models [31,32]. However, these effects can be observed depending on aspects such as anesthetic type, duration of surgery, genetic predisposition, and comorbidities, which should be considered to determine the safety of isoflurane and sevoflurane with respect to oxidative stress and inflammatory response in humans [8].

In addition, excess iron accumulates in the body, disrupting the normal function of mitochondria and numerous enzymes, worsening the body’s response to inflammation, and causing oxidative stress [33]. Additionally, excess iron leads to a number of neurodegenerative disorders such as Parkinson’s, Alzheimer’s, and Friedrich’s ataxia; it damages liver, heart, pancreas, and CNS; and it leads to endocrine dysfunction [34,35]. However, controversial results of the inflammatory response to iron status have been reported, with some claiming that moderate levels of dietary or intraperitoneal iron intake have an anti-inflammatory effect by triggering the M2 phenotype expression in macrophages to reduce the proinflammatory response. On the other hand, systemic excess iron supports the inflammatory response by triggering the M1 macrophage population [36].

Therefore, the aim of this study was to investigate the effect of inhaled anesthetics, isoflurane, and sevoflurane individually and in the presence of iron on the prooxidative and/or antioxidant effects of peripheral tissues and organs in chronic inflammation. Additionally, it was to find out if anesthetics can alter cell metabolism with respect to the transition metal as an initiator of ROS and inflammation, as well as of macrophage polarization.

A good indicator of inflammation, toxicity, and metabolic changes is the change in animal body weight and relative organ weights. The results indicate a loss of body weight, which was observed in all treated groups and was most pronounced in the group treated with sevoflurane (−3%), compared to the control group (+13%) (Figure 1). Weight loss in all groups exposed to one of the anesthetics is considered to be due to the effect of anesthetics on the decline in CNS metabolic activity and consequently on deficits in movement and food intake as long as the awakening and recovery phase lasts [37]. Additionally, weight loss in anaesthetized animals may be due to nausea, the disturbance of the circadian rhythm, and hypoglycemia and hypothermia, which can negatively influence the recovery period. Likewise, isoflurane has more incidences of mild airway hyper-reactivity when compared to sevoflurane, while complications such as bradycardia and hypotension, mild metabolic acidosis, and nausea were more with sevoflurane [38]. In addition, Orliaguet et al. [20] found that as postnatal age increased, the MAC value of sevoflurane and isoflurane increased significantly, reaching a peak in 9-day-old rats, after which it decreased. According to their research, the MAC value in adult rats (10–12 weeks) for isoflurane is 1.12% (1.07–1.18%) and sevoflurane 1.97% (1.84–2.10%) [20]. Therefore, weight loss in the 2.4 vol % sevoflurane group (Figure 1) can be explained as above-mentioned and due to constant treatment of the animals. It should certainly be considered that a decrease in body weight also occurred due to stress during constant treatment of animals every other day for a total of 28 days. On the other hand, weight loss in animals treated with iron dextran is possible due to diarrhea because excess iron might impair absorption of nutrients, can cause changes in intestinal enzymes that occur at the metabolic level, can influence enzyme synthesis, and can cause hormonal imbalances associated with excess iron [39]. Furthermore, the liver as well as the kidneys and spleen have many functions in the body, and their damage (either diseases or toxins) can affect various organ systems and the basic metabolism of all cells. Therefore, measuring the relative weight of liver, lungs, kidney, and spleen is one of the most sensitive indicators of drug and compound toxicity. In the groups treated with iron dextran and iron dextran + sevoflurane, higher weights of liver, spleen, and kidney were observed compared to other groups (Figure 2a,b). According to the results, it seems that the number of macrophages of the reticuloendothelial system is crucial for the capture of iron in the liver and spleen, as well as in the kidney, and it is assumed that this is probably due to an accumulation of macrophages in these tissues.

By injecting iron intraperitoneally, peritoneal macrophages are the first to come into contact with iron and phagocytose it [34]. Specifically, bound iron first accumulates in the tissue macrophage system of the liver, spleen, bone marrow, and other organs (phase of siderosis), and after filling the capacity of this system begins to be deposited in parenchymal cells of organs such as liver, heart, pancreas, and other glands with internal secretion, leading to their damage and dysfunction. The accumulation of iron in the reticuloendothelial system is an important defense response of the organism in preventing the occurrence of inflammation due to the presence of pathogens for which iron is crucial for their reproduction and spread [40]. On the other hand, a decrease in lung weight was observed in the groups treated with isoflurane and iron dextran + isoflurane, compared to the control group (Figure 2b). The explanation for the decreased lung weight is based on the mechanism of action of the anesthetic and the decrease in mucociliary function of epithelial cells leading to the accumulation of mucus during prolonged exposure to the anesthetic. The consequent airway obstruction leads to alveolar collapse and the development of atelectasis, and thus hypoxemia and respiratory infection [37]. The lower weight of other organs in the groups treated with isoflurane and iron dextran + isoflurane indicates possible hypoxia and consequent degradation of some cells of these tissues caused by anesthetics. Cytotoxicity caused by isoflurane has not been observed with short-term use of isoflurane, but long-term use indicates DNA damage in lymphocytes and other tissue cells [41,42].

Inhaled anesthetics significantly affect the respiratory system by reducing respiratory volume and increasing respiratory rate leading to rapid shallow breathing, decreased alveolar ventilation, and consequent systemic hypoxia. Respiratory depression is further stimulated by a reduced ventilation response to elevated levels of CO_2_ in the blood, i.e., a reduced ventilation response to hypoxia. At prolonged use of anesthetics, respiratory depression is further promoted by airway atelectasis. Although all anesthetics to some extent compensate for the resulting hypoxia by bronchodilation, isoflurane-induced airway irritation increases the degree of hypoxia compared to the sevoflurane [37]. In addition, Li et al. [43] demonstrated an increase in HIF-1α levels using isoflurane in the hepatocyte cell culture, Hep3B. Increased levels of HIF-1α in the body stimulate the production of erythropoietin in the liver and kidneys and the formation of erythrocytes, which is consistent with our results in the groups treated with isoflurane and iron dextran + isoflurane (Figure 3a).

Additionally, the analysis of haematological parameters confirms the presence of hypoxia and consequent increase in total erythrocytes in all groups, especially in groups treated with isoflurane and iron dextran + isoflurane (Figure 3a) where an increase in almost all hematological parameters is seen compared to other groups (Table 3). Increased erythropoiesis is also confirmed by elevated MCV (Table 3) in all groups, especially in the groups treated with isoflurane and iron dextran + isoflurane.

According to Li et al. [43], multiple signaling pathways directly activate genes responsible for systemic hypoxia in cells after isoflurane exposure. It is known that hypoxia is a hallmark trait of inflamed tissues, which is associated with leukocyte activation and inflammation and may act as major regulator of immune cell metabolic function (Figure 3b and Figure 4) [44]. The change in the metabolic function of activated cells is manifested in a reduced number of lymphocyte cells compared to iron dextran, which shows a slight degradation of cells and lymphocyte apoptosis after prolonged exposure. Isoflurane and the presence of hypoxia cause increased cell damage, leading to inflammation as evidenced by increased neutrophil counts compared to control (Figure 4b). On the other hand, the total number of leukocytes did not increase significantly in the groups exposed to anesthetics (Figure 3b) due to the negative hypoxic microenvironment showing increased sensitivity to anesthesia. The presence of inflammation, however, shows an increased concentration of RANTES (Figure 7b), whose increase is crucial for the migration and recruitment of T cells, dendritic cells, eosinophils, NK cells, mast cells, and basophils and is mostly produced by macrophages, which strengthen the inflammatory process in damaged tissue [45]. Total leukocyte counts were increased in the iron dextran-treated group and in the combination with anesthetics (Figure 3b), while RANTES levels were lower due to a sufficient number of inflammatory cells in the blood.

The presence of oxidative stress was confirmed by the analysis of biochemical parameters that indicate damage of the liver and other organs and increased levels of enzymes, proteins, metabolites, and substrates, as well as pathophysiological consequences of anesthetics and iron dextran. Such low ALP levels in the sevoflurane and iron dextran + isoflurane-treated groups (Table 1) may indicate malnutrition, absorption disorders, anemia, vitamin B12, B6, magnesium, and folic acid deficiency [46]. The groups treated with iron dextran and iron dextran + sevoflurane show the lowest levels of ALT and AMY, which may be due to severe intoxication of the liver and its damage, because of possible iron accumulation and ROS (Figure 8). Groups treated with isoflurane and the combination of iron dextran + isoflurane show higher blood glucose levels (Table 2), respectively, and hyperglycemia as a consequence of prevented exocytosis of insulin from pancreatic beta cells resulting from the harmful effects of isoflurane [47]. Low blood glucose concentrations in the group exposed to sevoflurane (Table 2) serve as another parameter that can confirm their possible malnutrition.

Furthermore, the kidney and liver damage result from impaired homeostasis between the antioxidant system and increased oxidative stress in cells induced by anesthetics and iron dextran. While higher activities of antioxidant enzymes (GSH, SOD, and CAT) indicate the presence of ROS, their low values are an indicator of depletion of the antioxidant system and are associated with damage and toxic effects of ROS. The release of GSH from cells has been observed in healthy hepatocytes and macrophages due to the role of maintaining antioxidant protection in the extracellular space, but its passive release and lower levels were observed immediately before cell death [48]. The greatest depletion of GSH, often associated with higher concentrations of MDA, is seen in the liver and kidney tissue after anesthetics and iron dextran administration, confirming their hepatotoxic and nephrotoxic effects and cell death by apoptosis (Figure 8 and Figure 9). In addition, Aydın et al. [49] confirmed that iron dextran causes lipid peroxidation with depletion of GSH antioxidants in the liver and cell death by apoptosis after iron accumulation. The potential nephrotoxic effect of iron dextran also supports the depletion of SOD enzyme activity (Figure 9c), suggesting the emergence of high concentrations of superoxide radicals [50]. Somewhat less depletion of GSH was also observed in the lung tissue in the iron-dextran + isoflurane-treated group (Figure 10d) as a response to hypoxia.

Macrophages play a key role in immunity and tissue homeostasis, protecting the organism from infection, as well as maintaining essential tissue-specific functions [51]. Macrophages also play an essential role in iron homeostasis by recycling iron, as a key element in the functioning of all cells and the maintenance of numerous functions in the body. In mammals, most of the iron used to produce hemoglobin is recycled after phagocytosis of aged red blood cells by macrophages, while the intake of iron from food absorbed through enterocytes is limited [52]. It is clear that the regulation of iron homeostasis can affect the immune function of macrophages and their polarization. The most important indicators of macrophage polarization and the progress of the inflammatory reaction are NO (Figure 5), Arg-1 (Figure 6), Th1/Th2 cytokine ratio (Figure 7a), and ROS levels.

M1 macrophages metabolize L-arginine to NO and citrulline via nitric oxide synthase, while M2 macrophages metabolize L-arginine to ornithine and urea via the enzyme arginase. The balance between L-arginine consumption by arginase and nitric oxide synthase determines the outcome of an inflammatory reaction in which higher NO concentrations indicate an increase in the inflammatory response, and higher Arg-1 activity indicates a delayed inflammatory response and tissue healing and reparation [53]. In anesthetic-treated groups it is found a mixed M1/M2 macrophage populations, and that indicate significantly higher levels of NO and Arg-1 activity in spleen tissue in combination-treated groups (Figure 5c and Figure 6b). A mixed population of macrophages is also present in kidney tissue in groups treated only with anesthetics (Figure 5b and Figure 6a), which indicates an inflammatory reaction caused by anesthetic metabolites, as well as a compensatory response of macrophages to tissue repair and remodulation. The liver is dominated by the M1 macrophage population; NO levels and Th1 cytokine levels were increased in all groups, especially in the groups treated with sevoflurane, isoflurane, and iron dextran + sevofuran (Figure 5a).

The ratio between IL-12 and IL-10 best indicates the polarization of macrophages. The highest level of inflammatory cytokines in serum (IL-1β, IL-6, IFN-γ, and TNF-α) is visible in the group exposed to sevoflurane and less in the group exposed to isoflurane, which indicates M1 polarization (Figure 7a). IFN-γ is one of the key cytokines that stimulate M1 macrophage activity, triggers inflammation, and enhances macrophage activity as an antigen-presenting cell. On the other hand, groups exposed only to anesthetics show an increase in serum anti-inflammatory cytokines, mostly IL-10, confirming the compensatory or regulatory nature of anesthetics via a second signaling pathway by initiating the anti-inflammatory process (Figure 7a) as response to increased ROS levels and initiating the tissue remodeling process. Multiple signaling pathways appear to be involved in the response to increased levels of anesthetics. According to the obtained results, it seems that in the groups treated only with anesthetics, the proinflammatory M1 population of macrophages initially predominates, and later due to the damage and changed environmental conditions, other regulatory signals and tissue regeneration processes occur [53]. The group exposed to isoflurane shows the highest levels of GM-CSF (Figure 7a), confirming the cytotoxic effect of isoflurane on tissues [42]. GM-CSF together with IL-1 promotes enhanced stem cell differentiation in the direction of myeloid and erythroid lineages [54], and delays neutrophil apoptosis and their degradation during inflammatory reactions while maintaining their abundance (Figure 4a) [55]. The polarization of macrophages controls functionally divergent processes; one of these is nitric oxide (NO) production, which in turn is controlled in part by HIF factors. According to Takeda et al. [56], the HIF-α isoforms can be differentially activated: HIF-1α is induced by Th1 cytokines in M1 macrophage polarization, whereas HIF-2α is induced by Th2 cytokines during a M2 response. This differential response was most evident in polarized macrophages through HIF-α isoform-specific regulation of the inducible NO synthase gene by HIF-1α, and the arginase1 gene by HIF-2α. This clear difference in the level of NO and Arg1 in our study was observed, especially in the kidneys (Figure 5b and Figure 6a). According to that, two HIF-α isoforms act under the control of differing polarizing cytokines such as Th1 and Th2 signaling, and this signaling, unexpectedly using the HIF transcription factors, is key to regulating NO homeostasis.

A study by Agoro et al. [36] points out that the IL-12/IL-10 ratio can help to determine macrophage polarization, where their high ratio indicates the presence of an M1 population, while a lower ratio indicates an M2 population. Guided by this, a higher serum IL-12/IL-10 ratio in the iron dextran-treated group (Figure 7a) indicates the presence of an M1 macrophage population and indicates the presence of systemic iron-induced inflammation. These results are consistent with an increased ROS levels in the liver as well as increased liver damage and the incidence of nonalcoholic fatty liver disease (NAFLD). The group treated with the combination of iron dextran + sevoflurane does not show the expected elevated levels of proinflammatory cytokines (Figure 7), which confirms that under changed conditions, the presence of sevoflurane may trigger an anti-inflammatory response consistent with research by Gerber et al. [31]. Moreover, the authors suggest multiple regulation pathways of sevoflurane and its anti-inflammatory action under conditions of induced inflammation. In mentioned paper, the inflammatory reaction was caused by *Escherichia coli*, where the authors suggest that sevoflurane may inhibit NF-κB, but the second signaling pathway may activate a direct increase in NO, thereby promoting phagocytosis and bactericidal properties of peritoneal macrophages [31]. Furthermore, Agoro et al. [36] show that intraperitoneal injection of iron dextran under conditions of lipopolysaccharide-induced inflammation (LPS) in mice can elicit an anti-inflammatory effect. These papers clearly indicate that self-derived stimulating signals and environmental conditions affect macrophage heterogeneity and plasticity. Confirmation of macrophage plasticity and environmental conditions can be seen in the group treated with iron dextran + isoflurane, where increased levels of the inflammatory cytokine IFN-γ (Figure 7a); oxidative stress; and depletion of the antioxidant system caused by this combination in the liver (Figure 8), kidney (Figure 9), and lung (Figure 10) tissue was seen. However, by monitoring Th1/Th2 cytokine levels and RANTES levels, it is observed that a combination of iron dextran and anesthetic reduce blood cytokine levels according to control group values; more precisely, the polarization of macrophages goes according to the M2 phenotype and anti-inflammatory effect. Namely, the data show that hypoxia-induced polarization of macrophages according to the M2 phenotype modifies the inflammatory microenvironment by reducing the release of proinflammatory cytokines.

## 5. Conclusions

Summarizing the results, it is clear that there is multiple regulation and that more signaling pathways are involved in response to increased levels of anesthetics and iron dextran, and that deeper insight at the molecular level is needed to monitor expression of key genes in the regulation process of the liver cells as well as environmental conditions associated with macrophage polarization. In addition, the results clearly indicate that excessive levels of iron and anesthetics lead to increased tissue and organ damage and that the anesthesiologist should assess the risk, select an anesthetic, and adjust techniques for optimizing surgery to reduce multiple anesthetic applications. Furthermore, interprofessional teamwork is needed to reduce the repeated use of anesthetics, especially in children and elderly patients undergoing different surgical procedures that require the use of anesthetics. These different procedures should be combined to ensure that single anesthetic encounter is performed and to reduce toxicity as well as costs for both the hospital and families. Additionally, according to our results, it seems important to monitor the dose of iron before and after surgery, given its toxicity classified as corrosive and cellular, and anesthesiologists should screen patients who are at high-risk for excess iron. Investigations into the possible therapeutic use of antimetabolite and antioxidant drugs with chelating ability would seem to be of interest to reduce the toxic effects of iron and volatile anesthetics and may be used in the management of patients with excess iron as well as improve outcomes of patients with transfusion-dependent anemia. Moreover, we suggest substituting volatile anesthetics for other anesthetic routes (intravenous anesthesia or locoregional techniques) where possible, and searching for new and non-metabolizable anesthetic agents and to establish an international threshold limits of exposure, because this approach would reduce overexposure of medical workers to anesthetics and waste anesthetic gases.

## Figures and Tables

**Table 1:** Biochemical values of enzymes and proteins from the blood of rats treated with inhaled anesthetics and iron dextran and their combination.

Biochemical Parameters—Enzymes and Proteins (Mean ± SE)						
GROUPS ^a^	ALP (U/L)	ALT (U/L)	AMY (U/L)	ALB (g/L)	TP (g/L)	GLOB (g/L)
**Cont.**	150.00 ± 3.82	42.33 ± 1.48	670.67 ± 12.79	57.50 ± 0.99	71.67 ± 2.06	14.00 ± 0.89
**Sevo.**	123.67 ± 2.64	34.00 ± 0.73	708.00 ± 30.01	50.67 ± 0.92 **^,^^∆^	65.33 ± 0.92 ^∆^	14.67 ± 0.84
**Iso.**	154.67 ± 4.42	42.33 ± 1.17	671.00 ± 0.97	56.33 ± 0.76	72.67 ± 1.12	16.00 ± 0.97
**Fe-dex.**	164.33 ± 9.12 ^■^	31.00 ± 0.97 **^,^^∆^	538.67 ± 10.70	54.67 ± 0.42	72.33 ± 1.12	17.33 ± 1.38
**Fe-dex. + Sevo.**	132.67 ± 5.06	28.33 ± 1.73 **^,^^∆∆^	502.50 ± 8.28 **^,^^■■,^^∆∆^	52.50 ± 1.20	72.83 ± 1.47	19.17 ± 1.07 *
**Fe-dex. + Iso.**	118.00 ± 7.96	39.00 ± 0.73	561.00 ± 28.22	50.33 ± 0.21 **^,^^∆^	65.33 ± 0.92 ^∆^	15.00 ± 0.97

^a^ Rats (*n* = 10 per group) were treated with *ip* injection of iron dextran (50 mg/kg), exposed to 2.4 vol % sevoflurane inhalation and 1.3 vol % isoflurane inhalation, and exposed to a combination thereof where inhaled anesthetics were given 2 h after *ip* injection of iron dextran. Animals were treated every other day for 28 days. The results are expressed as the mean value of each experimental group ± SE. * Statistically significant compared to the control group (* *p* < 0.05; ** *p* < 0.01); ^■^ Statistically significant compared to the sevoflurane group (^■^ *p* < 0.05; ^■■^ *p* < 0.01); ^∆^ Statistically significant compared to the isoflurane group (^∆^ *p* < 0.05; ^∆∆^ *p* < 0.01). Other statistically significant changes exist in: ALP (U/L)—Fe-dex. + Iso. vs. Fe-dex. (*p* < 0.05). Abbreviations: *ip*, intraperitoneal; SE, standard error; ALP, alkaline phosphatase; ALT, alanine aminotransferase; AMY, amylase; ALB, albumin; TP, total protein; GLOB, globulin; Cont., control group; Sevo., sevoflurane group; Iso., isoflurane group; Fe-dex., iron dextran group; Fe-dex. + Sevo., group treated with iron dextran and sevoflurane; and Fe-dex. + Iso., group treated with iron dextran and isoflurane.

**Table 2:** Biochemical values of metabolites and substrates from blood of rats treated with inhaled anesthetics and iron dextran and their combination.

Biochemical Parameters—Metabolites and Substrates (Mean ± SE)							
GROUPS ^a^	BUN (mmol/L)	GLU (mmol/L)	PHOS (mmol/L)	CRE (μmol/L)	Ca^2+^ (mmol/L)	Na^+^ (mmol/L)	K^+^ (mmol/L)
**Cont.**	5.01 ± 0.12	13.47 ± 0.81	2.09 ± 0.15	30.00 ± 1.90	2.48 ± 0.03	136.50 ± 1.38	5.22 ± 0.11
**Sevo.**	6.63 ± 0.38 *	10.67 ± 0.55	2.04 ± 0.05	41.67 ± 5.35	2.52 ± 0.03	134.33 ± 0.76	5.17 ± 0.08
**Iso.**	6.10 ± 0.33	16.17 ± 0.39 ^■^	2.11 ± 0.15	38.67 ± 4.01	2.46 ± 0.01	139.67 ± 0.76 ^■^	6.67 ± 0.35 ^■^
**Fe-dex.**	6.07 ± 0.44	14.97 ± 0.44	2.34 ± 0.14	38.33 ± 2.01	2.56 ± 0.04	136.33 ± 0.76	4.80 ± 0.10 ^∆∆∆^
**Fe-dex. + Sevo.**	4.55 ± 0.34 ^■■^	14.93 ± 0.55	2.47 ± 0.11	44.17 ± 2.83	2.51 ± 0.10	136.67 ± 1.12	5.77 ± 0.15
**Fe-dex. + Iso.**	5.33 ± 0.33	19.37 ± 0.31 **^,^^■■■^	2.53 ± 0.08 ^■^	40.33 ± 2.08	2.53 ± 0.02	136.67 ± 0.56	6.33 ± 0.02

^a^ Rats (*n* = 10 per group) were treated with *ip* injection of iron dextran (50 mg/kg), exposed to 2.4 vol % sevoflurane inhalation and 1.3 vol % isoflurane inhalation, and exposed to a combination thereof where inhaled anesthetics were given 2 h after *ip* injection of iron dextran. Animals were treated every other day for 28 days. The results are expressed as the mean value of each experimental group ± SE. * Statistically significant compared to the control group (* *p* < 0.05; ** *p* < 0.01); ^■^ Statistically significant compared to the sevoflurane group (^■^ *p* < 0.05; ^■■^ *p* < 0.01; ^■■■^ *p* < 0.001); ^∆^ Statistically significant compared to the isoflurane group (^∆∆∆^ *p* < 0.001). Other statistically significant changes exist in: K^+^ (mmol/L)—Fe-dex. vs. Fe-dex. + Iso. (*p* < 0.001). Abbreviations: *ip*, intraperitoneal; SE, standard error; BUN, blood urea nitrogen; CRE, creatinine; GLU, glucose; PHOS, phosphorus; Cont., control group; Sevo., sevoflurane group; Iso., isoflurane group; Fe-dex., iron dextran group; Fe-dex. + Sevo., group treated with iron dextran and sevoflurane; and Fe-dex. + Iso., group treated with iron dextran and isoflurane.

**Table 3:** Values of other haematological parameters in rats treated with inhalation anesthetics and iron dextran and their combination.

Groups a	Haematological Parameters of Blood (Mean ± SE)					
	Haemoglobin g/L	Haematocrit %	MCV (fL)	MCH (pg)	MCHC (g/L)	RDW (%)
**Cont.**	130.33 ± 2.69	35.33 ± 0.56	54.33 ± 0.21	20.33 ± 0.21	370.67 ± 4.23	13.33 ± 0.42
**Sevo.**	132.33 ± 0.76	36.33 ± 0.21	55.33 ± 0.21	20.00 ± 0.00	361.00 ± 2.03	13.67 ± 0.21
**Iso.**	156.33 ± 1.65 **^,^^■■^	46.33 ± 0.56 ***^,^^■■^	56.00 ± 0.00 **	19.00 ± 0.00	339.00 ± 1.46 *	13.00 ± 0.00
**Fe-dex.**	143.67 ± 3.39	40.00 ± 0.97	55.00 ± 0.00	19.67 ± 0.21	359.33 ± 3.31	12.33 ± 0.21
**Fe-dex. + Sevo.**	154.67 ± 0.92 *^,^^■^	42.67 ± 0.56	55.00 ± 0.00	19.67 ± 0.21	360.33 ± 2.14	12.67 ± 0.21
**Fe-dex. + Iso.**	154.17 ± 2.27 *^,^^■^	46.83 ± 0.60 ***^,^^■■^	56.00 ± 0.00 **	18.17 ± 0.17 ***^,^^■■^	330.00 ± 0.82 ***^,^^■^	12.33 ± 0.21

^a^ Rats (*n* = 10 per group) were treated with *ip* injection of iron dextran (50 mg/kg), exposed to 2.4 vol % sevoflurane inhalation and 1.3 vol % isoflurane inhalation, and exposed to a combination thereof where inhaled anesthetics were given 2 h after *ip* injection of iron dextran. Animals were treated every other day for 28 days. The results are expressed as the mean value of each experimental group ± SE. * Statistically significant compared to the control group (* *p* < 0.05; ** *p* < 0.01; *** *p* < 0.001); ^■^ Statistically significant compared to the sevoflurane group (^■^ *p* < 0.05; ^■■^ *p* < 0.01). Other statistically significant changes exist in: MCHC (g/L)—Fe-dex. vs. Fe-dex. + Iso. (*p* < 0.05); and Fe-dex. + Sevo. vs. Fe-dex. + Iso. (*p* < 0.05). Abbreviations: SE, standard error; MCV, average erythrocyte volume; MCH, average content of haemoglobin in erythrocytes; MCHC, average haemoglobin concentration in erythrocytes; RDW, erythrocyte size distribution; Cont., control group; Sevo., sevoflurane group; Iso., isoflurane group; Fe-dex., iron dextran group; Fe-dex. + Sevo., group treated with iron dextran and sevoflurane; and Fe-dex. + Iso., group treated with iron dextran and isoflurane.

## Data Availability

Data is contained within this article and supplementary file.

## Supplementary Materials

The following supporting information can be downloaded at: https://www.mdpi.com/article/10.3390/antiox11040708/s1, Table S1: Experimental groups and treatment method of experimental animals.
