# Supplementary material for: Effects of Volatile Anaesthetics and Iron Dextran on Chronic Inflammation and Antioxidant Defense System in Rats
Source: Antioxidants (Basel). 2022 Apr 3;11(4):708. doi: 10.3390/antiox11040708 (PMC9025161; doi:10.3390/antiox11040708)
Supplement: Supplementary file 1 [file antioxidants-11-00708-s001.zip › antioxidants-1622202-supplementary.pdf]

Table S1. Experimental groups and treatment method of experimental animals

Groups	The treatment method of animals within the research			
	**Composition of the test solution**	**Dose**	**Implementation**	**Treatment period (days)**
1. Control	0.9% NaCl	0.5 mL every other day	*ip*	28 days
2. Sevoflurane	Sevoflurane, an inhaled anaesthetic	2.4% every other day	inhalation	28 days
3. Isoflurane	Isoflurane, an inhaled anaesthetic	1.3% every other day	inhalation	28 days
4. Iron dextran	50 mg/kg FeH_2O_4S in 0.5 mL aqua pro	0.5 mL every other day	*ip*	28 days
5. Iron dextran in combination with Sevoflurane	50 mg/kg FeH_2O_4S in 0.5 mL *aqua pro* in combination with Sevoflurane	0.5 mL of test solution in combination with 2.4% inhaled anaesthetic every other day	*ip* + inhalation	28 days
6. Iron dextran in combination with Isoflurane	50 mg/kg FeH_2O_4S in 0.5 mL *aqua pro* in combination with Isoflurane	0.5 mL of test solution in combination with 1.3% inhaled anaesthetic every other day	*ip* + inhalation	28 days
